# Supplementary material for: Mapping of HOCl-oxidized RNA identifies abasic sites as major damage and oxidation product of oxo8G
Source: Nat Commun. 2025 Nov 21;16:10251. doi: 10.1038/s41467-025-65108-0 (PMC12639000; doi:10.1038/s41467-025-65108-0)
Supplement: Supplementary file 1 — Supplementary Information [file 41467_2025_65108_MOESM1_ESM.pdf]

Supplementary Information

Mapping of HOCl-oxidized RNA identifies abasic sites as major damage and oxidation product of oxo^8G

Marlies Weber[#,a], Kasturi Raorane[#,b], Clara Johanna Grampp[a], Valérie Bourguignon[b,c], Lea-Marie Kilz[a], David Glänzer[d], Virginie Marchand[c], Christoph Kreutz[d], Yuri Motorin*[,b,c] and Mark Helm*[,a]

a Johannes Gutenberg-University Mainz
Institute of Pharmacy and Biomedical Sciences
Staudingerweg 5
55128 Mainz, Germany
E-mail: mhelm@uni-mainz.de

b Université de Lorraine
CNRS, IMoPA UMR7365
F-54000 Nancy, France
E-mail: yuri.motorin@univ-lorraine.fr

c Université de Lorraine
CNRS, INSERM, UAR2008 IBSLor Epitranscriptomics and RNA Sequencing Core Facility
F-54000 Nancy, France

d Institute of Organic Chemistry and Center for Molecular Biosciences Innsbruck (CMBI)
University of Innsbruck
Innrain 80/82
6020 Innsbruck, Austria

# These authors contributed equally to this work.

* Corresponding authors

Supplementary Table 1. Abbreviations of nucleosides and nucleotides used throughout the manuscript.

Nucleoside	Abbreviation
cytidine	C
uridine	U
guanosine	G
adenosine	A
5-hydroxycytidine	ho^5C
5-chlorocytidine	Cl^5C
5-hydroxyuridine	ho^5U
5-chlorouridine	Cl^5U
8-oxoadenosine	oxo^8A
8-chloroadenosine	Cl^8A
8-oxoguanosine	oxo^8G
8-chloroguanosine	Cl^8G
5-carboxamido-5-formamido-2-iminohydantoin	r2Ih
diiminoimidazole	rDiz
guanidinohydantoin	rGh
imidazolone	rIz
spiroiminodihydantoin	rSp
oxazolone	rZ
abasic site	AP
5'-ribose phosphate	5'-RP
8-oxoguanosine monophosphate	oxo^8GMP

Supplementary Table 2. Nomenclature of modified nucleobases and nucleosides in DNA and RNA. In the realm of oxidation processes, a notable matter arises from the lack of a uniform nomenclature of modified nucleosides in DNA and RNA. To address this, we decided to employ the shared style for the abbreviation of modified e.g. methylated nucleosides. In the field of DNA research, it is common to refer to nucleobases when discussing modifications, while RNA scientists typically refer to nucleosides to ensure a clear distinction between DNA and RNA. Consequently, deoxyribonucleosides are specifically emphasized. Based on these considerations, the above conventions have been widely accepted.

Nomenclature	Common name	Abbreviation	Structure
nucleobase	8-oxoguanine	8oxoG	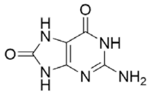
ribonucleoside	8-oxoguanosine	oxo^{8}G	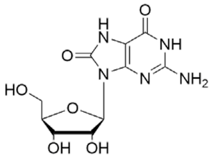
deoxyribonucleoside	8-oxo-deoxyguanosine	oxo^{8}dG, also known as OG	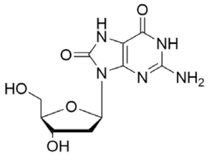

Supplementary Table 3. Resulting mass transitions of alternative guanosine oxidation products of the ^{12}C-, ^{13}C- and ^{15}N-labeled isotopes.

Compound	Precursor Ion	Neutral loss
r2Ih ^{12}C	318	132
r2Ih ^{13}C	328	137
r2Ih ^{15}N	323	132
rDiz ^{12}C	244	132
rDiz ^{13}C	252	137
rDiz ^{15}N	249	132
rGh ^{12}C	290	132
rGh ^{13}C	299	137
rGh ^{15}N	295	132
rIz ^{12}C	245	132
rIz ^{13}C	253	137
rIz ^{15}N	249	132
rSp ^{12}C	316	132
rSp ^{13}C	326	137
rSp ^{15}N	321	132
rZ ^{12}C	263	132
rZ ^{13}C	271	137
rZ ^{15}N	267	132

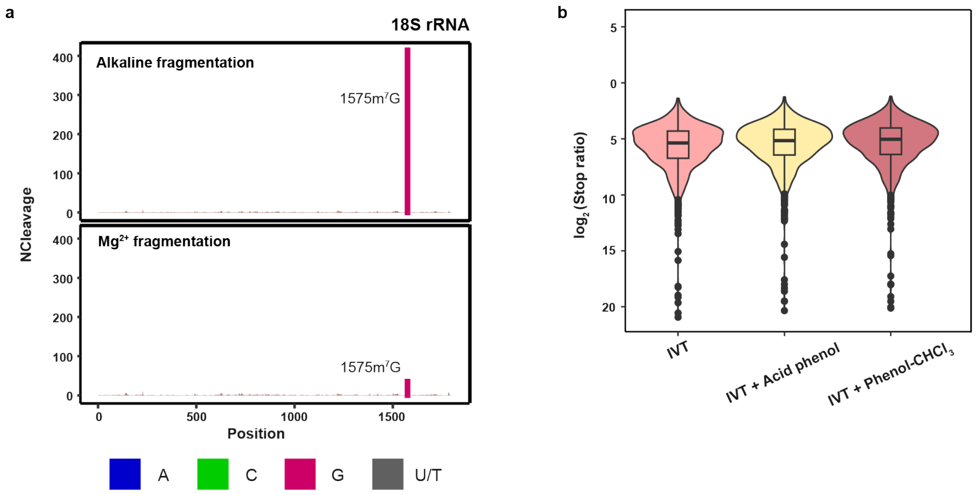

Supplementary Figure 1. Validation aspects of AAS on rRNA. A Sequencing profiles of native *S. cerevisiae* 18S rRNA following alkaline (top panel) and Mg^{2+} fragmentation (bottom panel). The natively present m^7G at position 1575 results in a strong signal shown as normalized cleavage (NCleavage) representing the number of reads starting at that position related to the total number of reads. The graph shows one sample from three biological replicates (n = 3). **B** Box and violin plots represent stop ratios (ratio of reads starting at that position to total number of passing reads) of the *S. c.* 18S rRNA *in vitro* transcript (IVT, 1460 data points) either sequenced directly after synthesis, after acid phenol extraction (IVT + Acid phenol, 1460 data points, p-value = 1.65 * 10^{-7}) or after phenol-chloroform extraction (IVT + Phenol-$CHCl_3$, 1460 data points, p-value = 5.10 * 10^{-4}). Box plots show the median (centre line), 25th and 75th percentiles (bounds of box), and whiskers represent minima and maxima. The experiment was performed in n = 2 biological replicates. Source data are provided as a Source Data file.

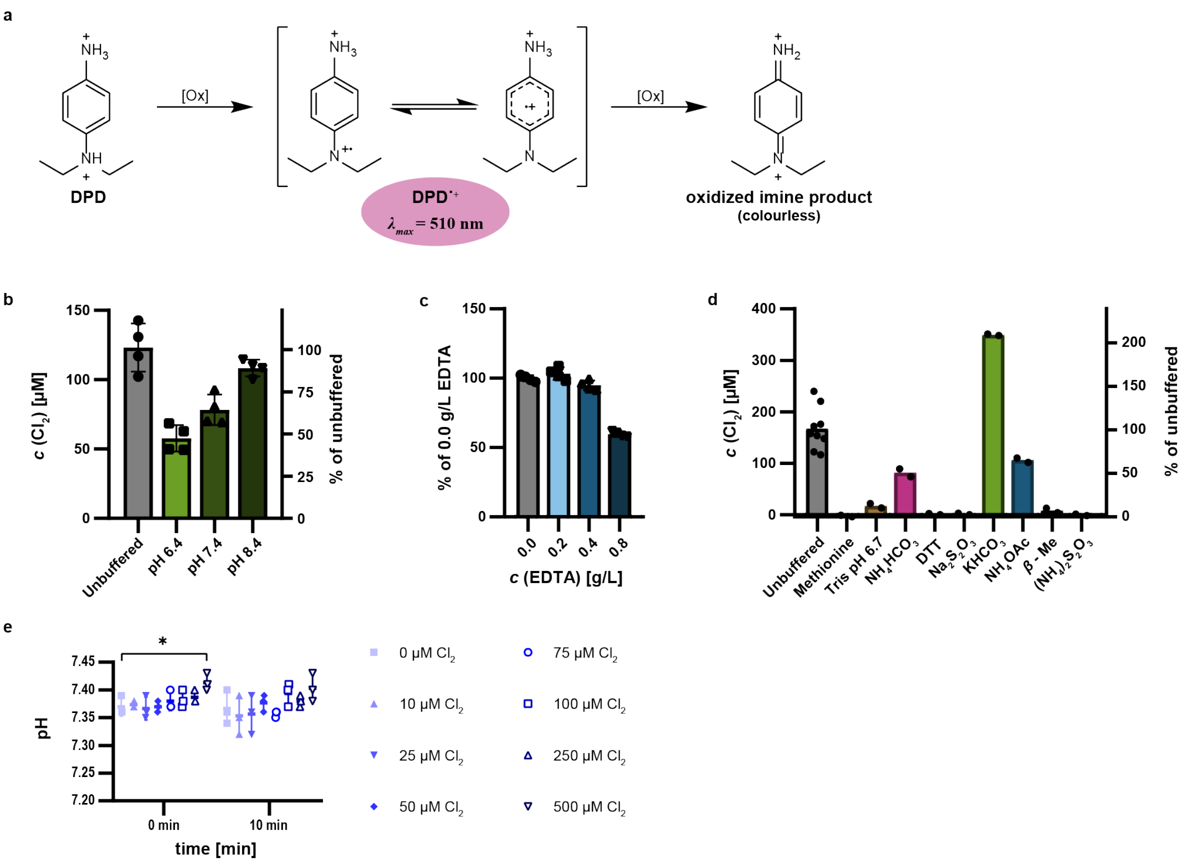

Supplementary Figure 2. Determination of free chlorine in HOCl using the DPD (*N*,*N*-dialkyl-1,4-phenylenediamine) method. Quantification was performed by adding HOCl to respective samples followed by the final addition of a DPD solution. Absorbance was measured directly at 510 nm in a 1 mL cuvette using a Jasco V-650 UV spectrophotometer. **a** Molecular structures of reduced DPD, its oxidized pink-colored form $DPD^{\bullet +}$ and the colorless oxidized imine product. Quantification is based on the absorbance of the $DPD^{\bullet +}$ at 510 nm. **b** The determined amount of free Cl_2 was compared at different pH values (6.4, 7.4 and 8.4) of a phosphate-buffered solution and unbuffered ultrapure water. The left *y*-axis indicates absolute Cl_2 concentrations and the right *y*-axis shows relative values normalized to the unbuffered control. **c** Influence of different EDTA concentrations on the determined Cl_2 content shown as a proportion of a solution without EDTA. Free Cl_2 is indicated as a percentage of 0 g/L EDTA. **d** Comparison of different quenching agents (methionine, Tris buffer pH 6.7, ammonium bicarbonate NH_4HCO_3, dithiothreitol DTT, sodium thiosulfate $Na_2S_2O_3$, potassium bicarbonate $KHCO_3$, ammonium acetate NH_4OAc, *β*-mercaptoethanol *β*-Me, ammonium thiosulfate $(NH_4)_2S_2O_3$) with respect to the determined Cl_2 content. The left *y*-axis indicates absolute Cl_2 concentrations and the right *y*-axis shows relative values normalized to the unbuffered control. The concentrations of quenching agents were 10 mM for all reagents except for NH_4OAc, which was 500 mM, as used for ethanol precipitation. Data are presented as the mean of biological replicates ± SD, n = 9 for unbuffered conditions, n = 2 for other components. **e** pH analysis of oxidation experiments including phosphate buffer and HOCl in concentrations between 0 µM and 500 µM Cl_2. An ordinary one-way ANOVA (Dunnett's multiple comparisons test) was conducted using GraphPad prism 8.0 comparing 0 µM Cl_2 vs. 500 µM Cl_2 a significant difference was calculated (p = 0.0295). Each sample was measured at 0 minutes and after a 10-minute incubation period. Data are presented as the mean of three biological replicates ± SD, n = 3 unless stated otherwise. Source data are provided as a Source Data file.

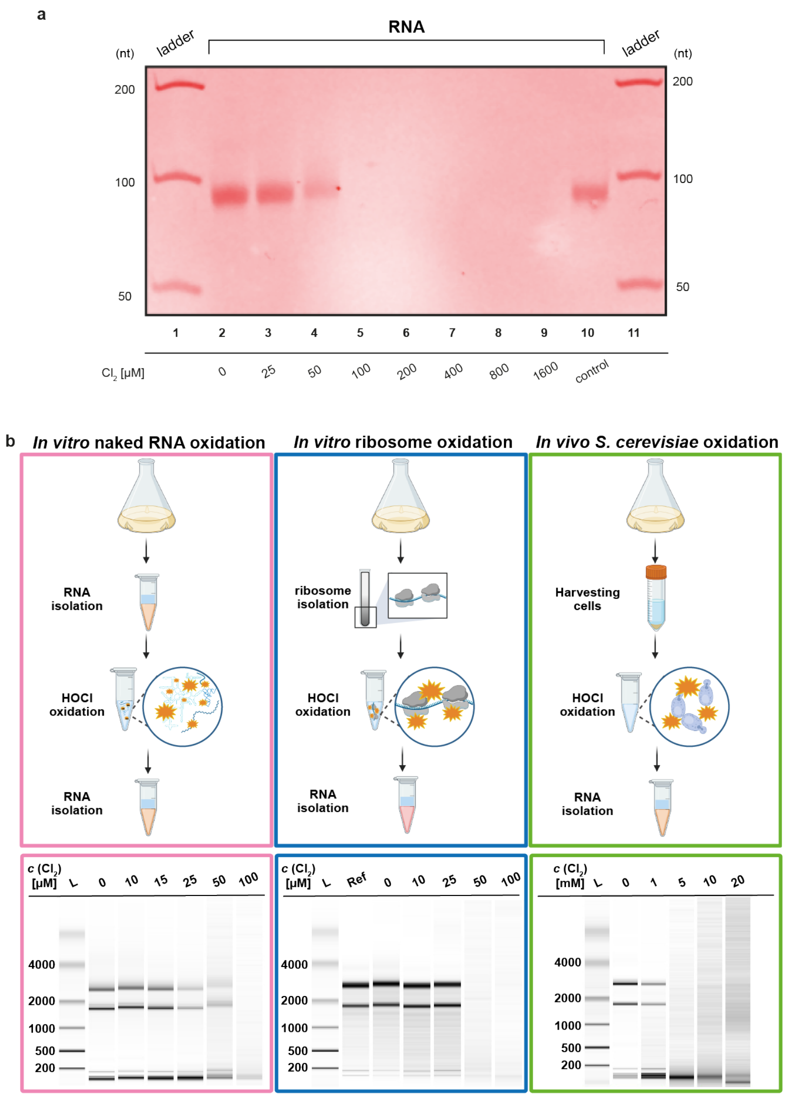

Supplementary Figure 3. RNA integrity of tRNA^{Asp} and *S. cerevisiae* RNA oxidized under different conditions. A Denaturing PAGE analysis of an *in vitro* transcript of tRNA^{Asp} (IVT-tRNA^{Asp}) in an HOCl oxidation series. Cl_2 concentrations range from 0 to 500 µM (lane 2 to 9). Lane 1 and 11 contain ladders indicating 50, 100 and 200 base pairs. Lane 10 contains an untreated control. 100 ng was loaded for each sample and samples were visualized using GelRed™ staining. The experiment was performed in biological replicates (n = 3). **B** HOCl oxidation was performed under 3 experimental conditions: *in vitro* on isolated total RNA (pink, left panel), *in vitro* upon isolation of ribosomes by sucrose gradient (blue, middle panel) and *in vivo* after harvesting cells and resuspending them in PBS buffer prior to oxidation with HOCl (green, right panel). RNA integrity of oxidized samples was analyzed using capillary electrophoresis after RNA isolation and purification. The resulting profiles are shown for one representative oxidation series. The left lane shows a ladder (L) followed by an untreated control and the oxidized samples (Cl_2 concentrations are indicated at the top of each lane). The ribosome profile additionally includes a reference of a stock sample (Ref). The graphs show one sample from three biological replicates (n = 3) for the *in vitro* naked RNA and *in vivo* oxidized samples and n = 2 for ribosomes. Source data are provided as a Source Data file. Created in BioRender. Raorane, K. (2025) https://BioRender.com/y1wc7b9

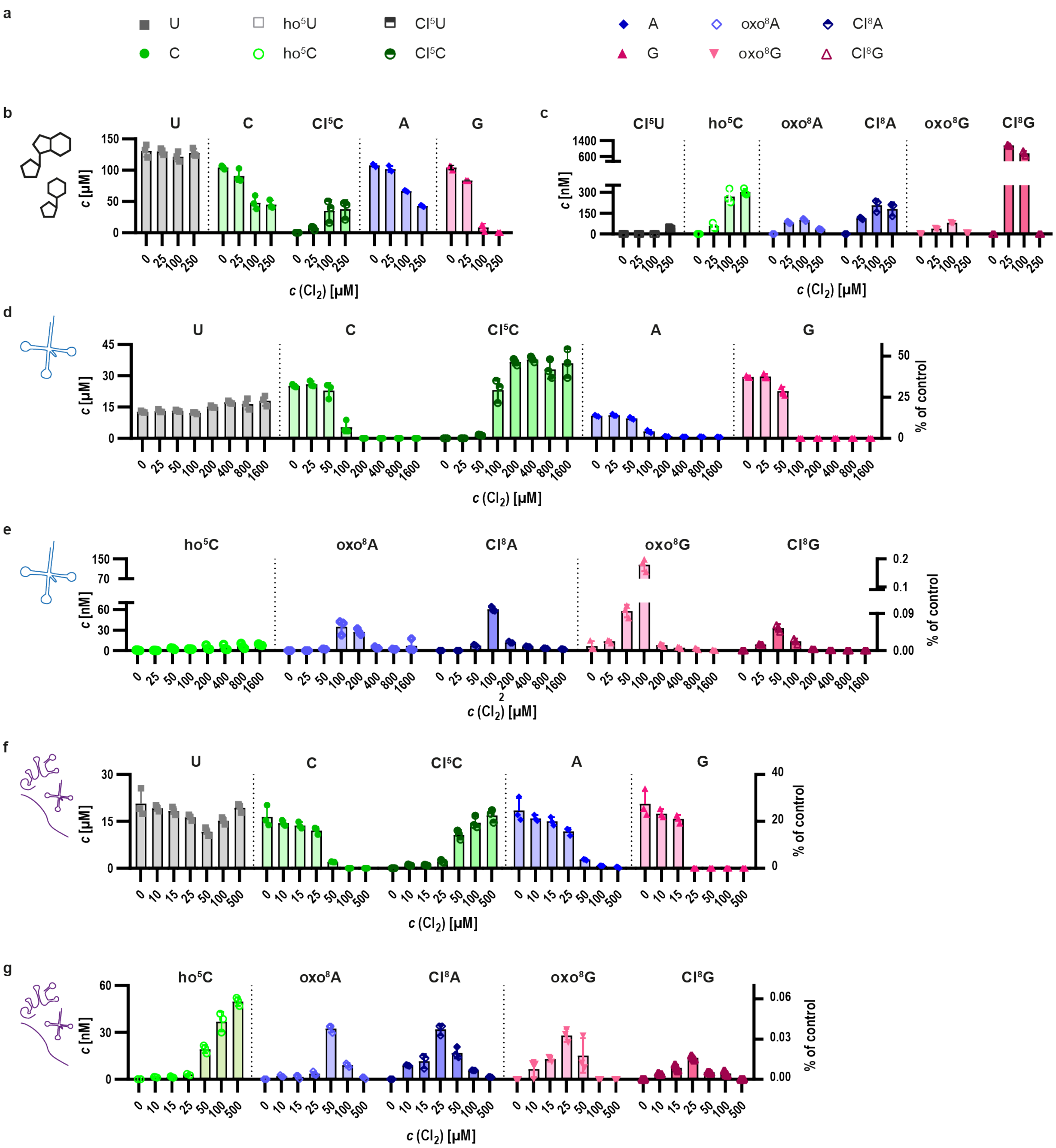

Supplementary Figure 4. LC-MS/MS quantification of oxidation series performed on mononucleosides, IVT-tRNA^{Asp} and *in vitro* oxidized total *S. cerevisiae* RNA. Nucleosides and their oxidation products were quantified as absolute amounts using external standards for calibration by LC-MS/MS. Shown are mean values of biological replicates ± SD, *n* = 3. **A** Color code for the displayed nucleosides. **B** Absolute quantification of uridine, cytidine, adenosine and guanosine after HOCl oxidation of mononucleosides. Concentrations are indicated in µM relative to the initial reaction volume. **C** Absolute amounts of the corresponding oxidation products present in nM concentrations. **D** Absolute quantification of a HOCl concentration series on IVT-tRNA^{Asp}. Cl_2 concentrations ranged from 0 to 1600 µM and are indicated on the *x*-axis. The left *y*-axis indicates nucleoside concentrations in µM and the right *y*-axis shows relative proportions normalized to total nucleosides present in the untreated control. **E** Absolute amounts of the oxidized nucleosides indicated in nM on the left *y*-axis. The right *y*-axis is normalized to the total of nucleosides in the untreated control. **F** and **G** show the absolute amounts of *ex vivo* oxidized total *S. c.* RNA in µM (**F**) and nM (**G**) concentrations according to the analysis shown for IVT-tRNA^{Asp}. Note that the apparent increase in pyrimidines at high concentrations of Cl_2 results from a bias in UV normalization as a consequence of purine chromophore oxidation. Source data are provided as a Source Data file.

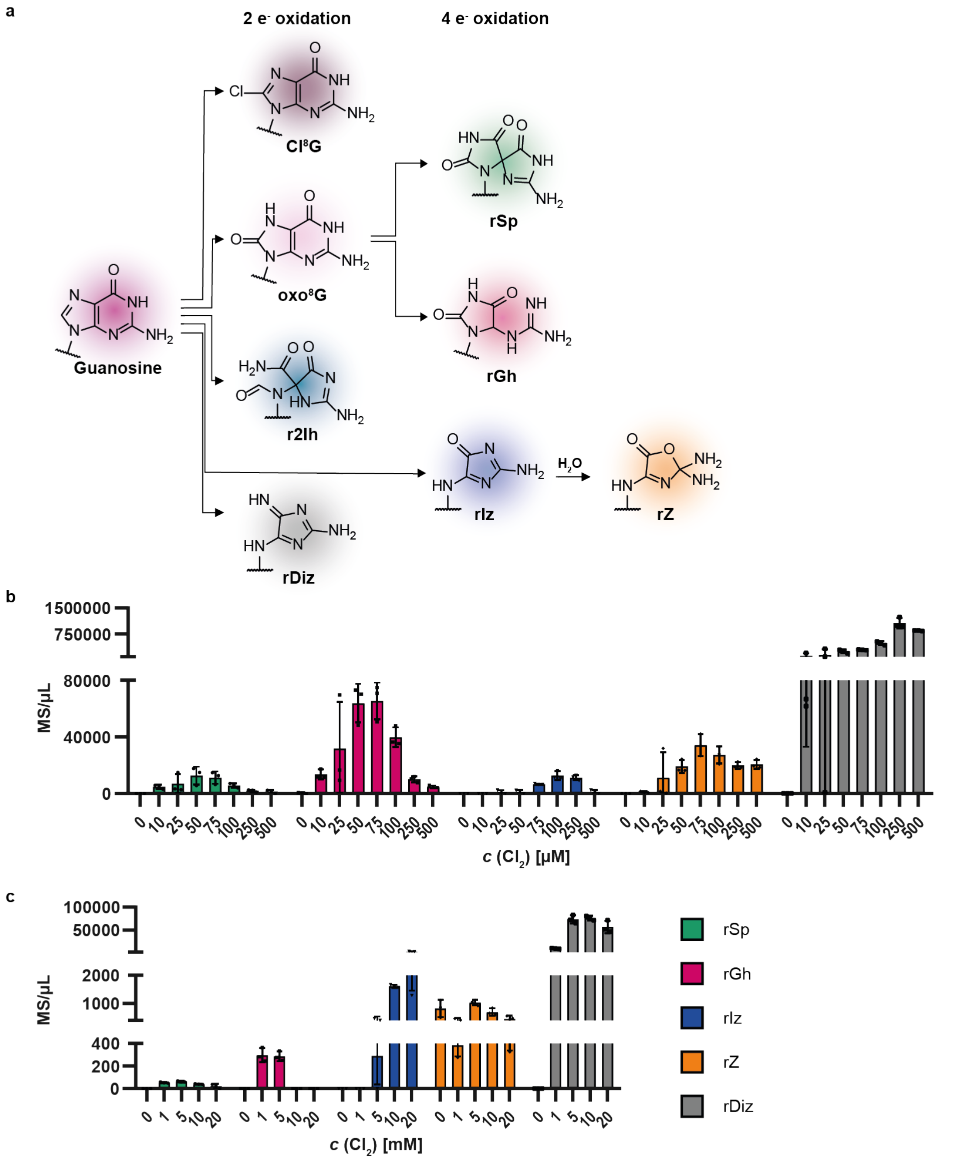

Supplementary Figure 5. Guanosine oxidation products. A C8 chlorination and oxygenation products of 2 e^- oxidation reaction are 8-chloroguanosine (Cl^8G, dark pink) and 8-oxoguanosine (oxo^8G, light pink), which can be further oxidized by a second 2 e^- oxidation reaction leading to ring-opening. The resulting products are spiroiminodihydantoin (rSp, green) or guanidinohydantoin (rGh, pink) depending on reaction conditions. C5 oxidation reaction leads to diiminoimidazole (rDiz, gray), 5-carboxamido-5-formamido-2-iminohydantoin (r2Ih, cyan) and imidazolone (rIz, dark blue), which is sensitive towards hydrolysis resulting in oxazolone (rZ. orange). **B** The MS signals of the ring-opened oxidation products, as illustrated in A, have been normalised to the initial reaction volume of a HOCl oxidation series performed on an unmodified 38mer. **C** The MS signals of the ring-opened oxidation products, as illustrated in A, have been normalised to the initial reaction volume of a HOCl oxidation series performed *in vivo* on *S. c.* cells. Shown are mean values of biological replicates ± SD, $n = 3$. Source data are provided as a Source Data file.

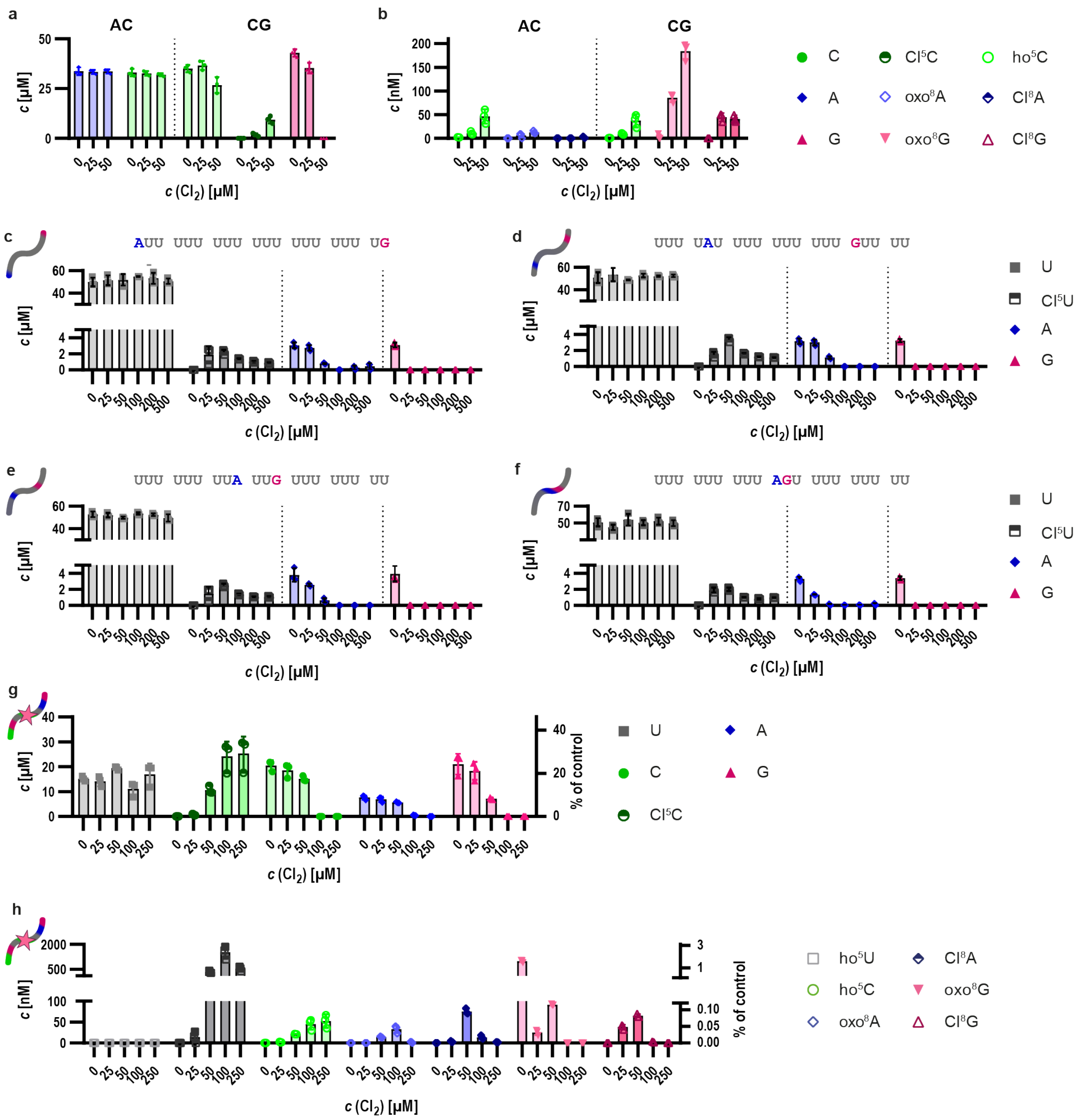

Supplementary Figure 6. Absolute LC-MS quantification of HOCl oxidation series experiments performed on synthetic oligoribonucleotides. A, B Absolute concentrations of nucleosides from oxidized binary oligonucleotides containing either A and C or C and G. Oxidation was performed in the range from 0 to 500 µM Cl_2 and quantified by LC-MS/MS using external standards for calibration purposes. **A** Concentrations of parent nucleosides A in blue, C in green, Cl^5C in dark green and G in pink are indicated in µM on the *y*-axis for each Cl_2 concentration. **B** Display of low abundant oxidation products in nM concentrations ho^5C in light green, oxo^8A in light blue, Cl^8A in dark blue, oxo^8G in light pink and Cl^8G in dark pink. **C - F** LC-MS quantification results for four different oligonucleotides containing one A and one G within U stretches. Concentrations are given in µM in dependence of the applied Cl_2 concentration. **G, H** Absolute quantification of nucleosides from an oxidation series (0 - 500 µM Cl_2) of the oxo^8G-containing 38mer. Data are plotted in µM concentrations (**G**) for abundant nucleosides and in nM for low-abundant oxidized nucleosides (**H**) on the left *y*-axis in relation to the applied Cl_2 concentration on the *x*-axis. The proportion relative to the total of all nucleosides present in the untreated control is indicated on the right *y*-axis. All data are presented as mean values of three biological replicates ± SD (*n* = 3). Source data are provided as a Source Data file.

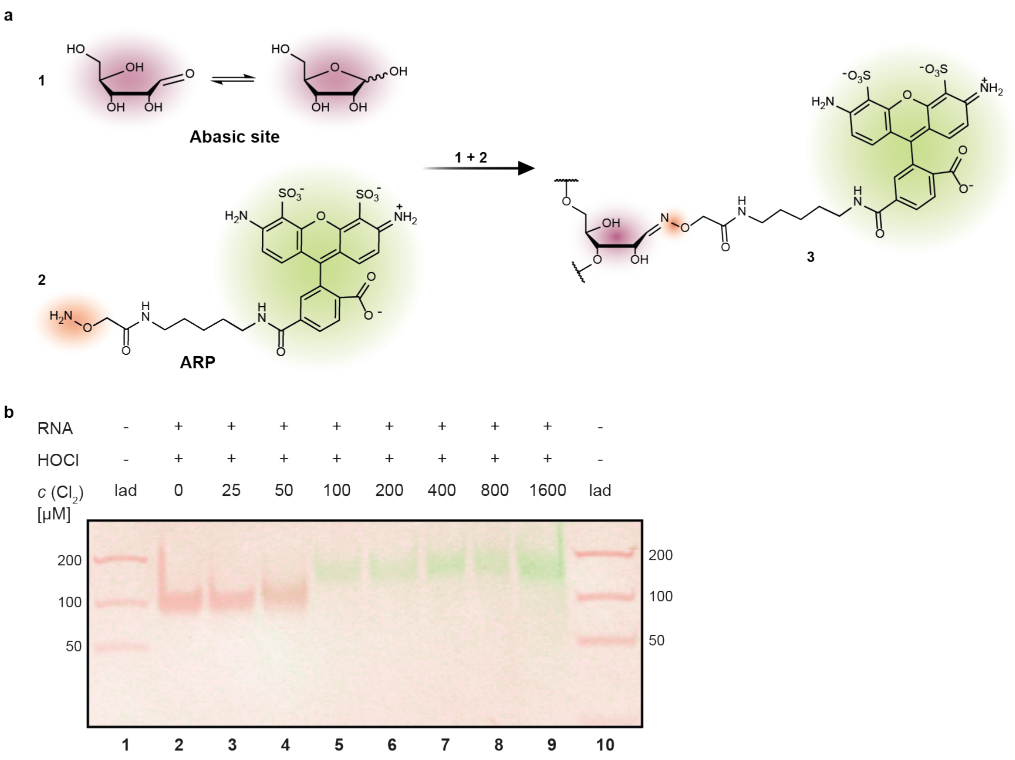

Supplementary Figure 7. Detection of abasic sites in oxidized RNA using an aldehyde-reactive probe (ARP). A The reaction scheme depicts the reaction between an abasic site **1** (pink) reacting and an aldehyde-reactive probe (ARP) **2**, which contains a fluorescent dye (green) and a nucleophilic hydroxylamine moiety (orange). This leads to the formation of a covalently linked, fluorescently labeled abasic site (**3**). **B** A 10% denaturing PAGE of an IVT-$tRNA^{Asp}$ that was oxidized in a range of Cl_2 concentrations from 0 µM to 1600 µM Cl_2 prior to ARP labelling. Lane 1 and 10 contain ladders indicating 50, 100 and 200 base pairs, lanes 2 to 9 contain the samples after oxidation, labelling and purification. Red bands correspond to GelRed signals (E_{Ex} = 532 nm, E_{Em} = 610 nm) and green bands show the fluorescent signal originating from successfully labelled abasic sites (E_{Ex} = 488 nm, E_{Em} = 526 nm). The graph shows one sample from three biological replicates (*n* = 3). Both images were edited and merged using ImageJ. Source data are provided as a Source Data file.

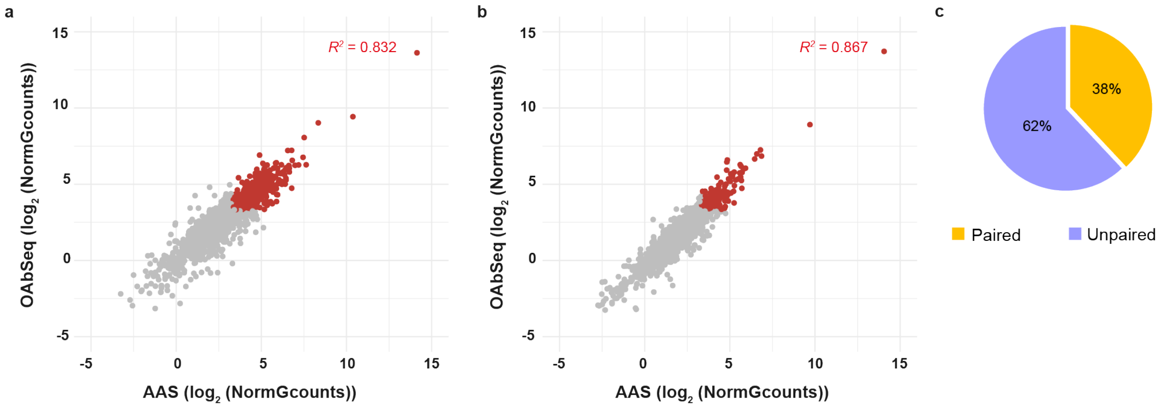

Supplementary Figure 8. Comparable oxidation of *S. cerevisiae* naked rRNA *in vitro* or in isolated ribosomes using both AAS and OAbSeq. A Correlation plot comparing AAS and OAbSeq scores of *S. cerevisiae* RNA oxidized *in vitro* (naked RNA) with 10 µM Cl_2. Sites that are three times above noise level are colored in red. Gray dots represent sites where either AAS, OAbSeq, or both scores fall below the specified threshold (NormGcounts = 10). Shown are data points of 1490 Gs in rRNA from biological replicates (*n* = 3). **B** Correlation plot comparing AAS and OAbSeq scores of *S. cerevisiae* ribosomes oxidized *in vitro* with 25 µM Cl_2. Sites that are three times above noise level are colored in red. Gray points represent sites where either AAS, OAbSeq, or both scores fall below the specified threshold (NormGcounts = 10). Shown are data points of 1490 Gs in rRNA from biological replicates (*n* = 2). **C** Proportions of unpaired and paired guanosines oxidatively damaged in 25S, 5S, 5.8S and 18S rRNA following treatment with HOCl under *in vitro* ("naked" RNA) experimental conditions. The 2D structure of *S. cerevisiae* rRNA was compared using dot-bracket notation given by the ncRNA database called RNAcentral (https://rnacentral.org/). Source data are provided as a Source Data file.

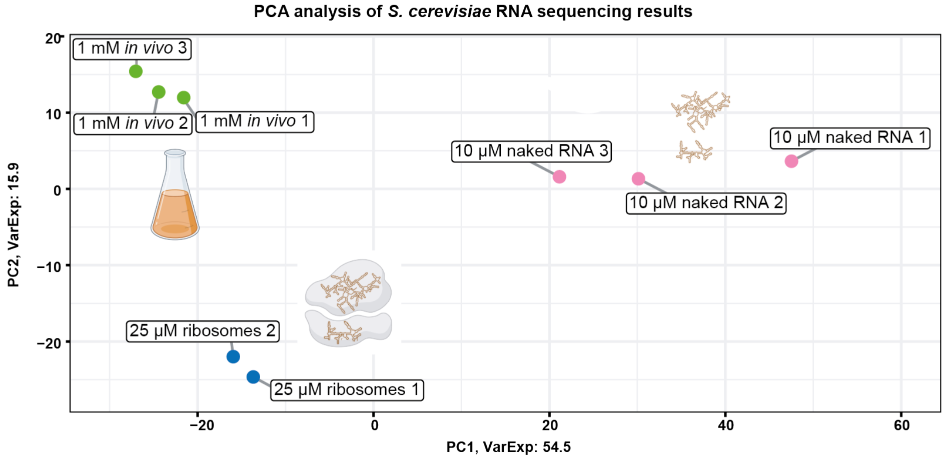

Supplementary Figure 9. PCA analysis of oxidized *S. cerevisiae* rRNA from different preparations. *S. cerevisiae* rRNA was oxidized and analyzed using AAS (see also methods section) under three conditions. Naked RNA (pink): total RNA extracted from yeast was oxidized *in vitro* with 10 µM Cl_2. Ribosomes (blue): ribosomal complexes were isolated by sucrose gradient centrifugation from lysed yeast, oxidized *in vitro* with 25 µM Cl_2 and RNA subsequently isolated. *In vivo* (green): cultured yeast cells were exposed to 1 mM Cl_2 followed by total RNA extraction. All isolated RNA samples were subjected to AAS and oxidized sites compared across the three conditions. Principal component analysis (PCA) of *in vivo* (*n* = 3), ribosomal *(n* = 2) and naked rRNA (*n* = 3) samples revealed a clear separation of naked RNA from ribosomal and *in vivo* conditions. PCA analysis was performed including 1436 data points for each condition. Source data are provided as a Source Data file. Created in BioRender. Gonnet, J. (2025) https://BioRender.com/j5j4iue, https://BioRender.com/pq8bl4r.

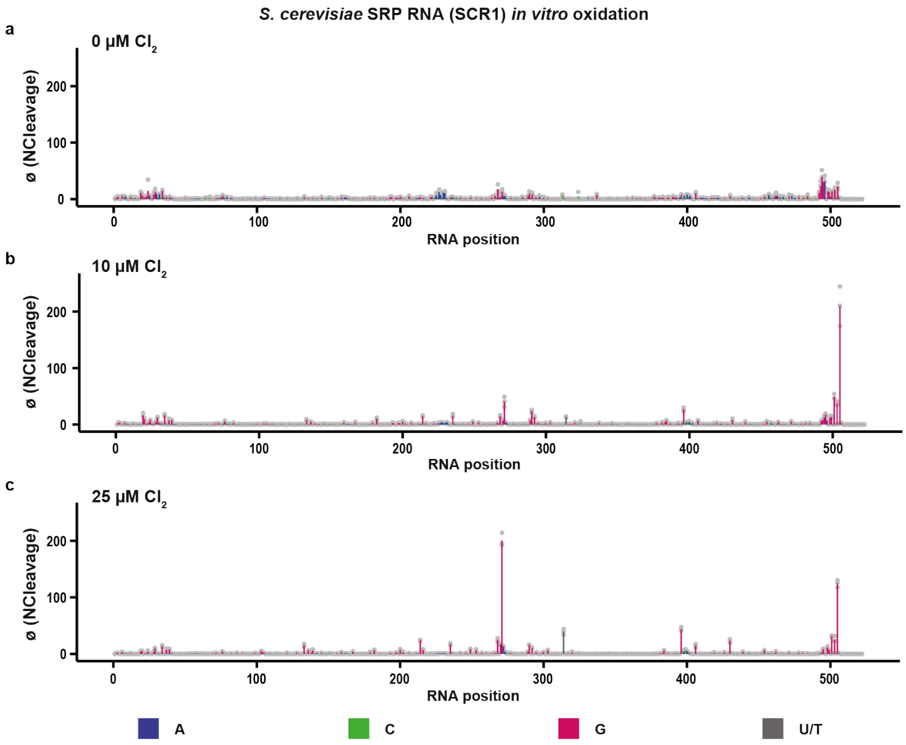

Supplementary Figure 10. AAS profile of *S. cerevisiae* SCR1 upon *in vitro* oxidation. A - C AAS profiles of *S. cerevisiae* SRP RNA (SCR1) showing the average of normalized cleavage counts (NCleavage, *y*-axis) for all RNA positions (*x*-axis). **A** The profile of an untreated control (0 µM Cl_2) reveals no signals above background level. Signals appear after oxidation using 10 µM Cl_2 (**B**) and 25 µM Cl_2 (**C**) preferentially at G residues such as G271. Shown are mean values of three biological replicates (n = 3). Source data are provided as a Source Data file.

Materials

Supplementary Table 4

LC-MS standard	Supplier
5-hydroxycytidine-5'-triphosphate (ho^{5}CTP)	tebubio, Germany
5-chlorocytidine (Cl^{5}C)	BIOLOG, Germany
5-hydroxyuridine (Cl^{5}U)	Biosynth Ltd, UK
5-chlorouridine (Cl^{5}U)	Santa Cruz Biotechnology, USA
8-oxoguanosine (oxo^{8}G)	BIOSYNTH CARBOSYNTH, UK
8-chloroguanosine (Cl^{8}G)	BIOLOG, Germany
8-oxoadenosine (oxo^{8}A)	abcr, Germany
8-chloroadenosine (Cl^{8}A)	BIOLOG, Germany
D-ribose 5-phosphate disodium salt (5'-RP)	Sigma-Aldrich, Germany

Supplementary Table 5

Oligomer	Sequence	Supplier
T7 forward primer	CGCGCGAAGCTTAATACGACTCACTATA	Biomers, Ulm, Germany
human $tRNA^{Asp}$ PCR template	TGGCGGGCCGTCGGGGAATCGAACCCCGGTCTCCCGCGTGA CAGGCGGGGATACTCACCACTATACTAACGACCCTATAGTG AGTCGTATT	IBA Lifesciences, Goettingen, Germany
T7 reverse primer $tRNA^{Asp}$	TGGCGGGCCGTCG	IBA Lifesciences, Goettingen, Germany
GU binary oligomer	GGUUGUGGUGGUUUGUUGGU	Biomers, Ulm, Germany
CU binary oligomer	UUCUUUCUUUCCCUUCCUUU	Biomers, Ulm, Germany
AU binary oligomer	UUAUAUUUAUAAAUAUUAAA	Biomers, Ulm, Germany
AG binary oligomer	AAAGGAAGGGAAAGAAAGAA	Biomers, Ulm, Germany
AC binary oligomer	ACCAACAAACCACCACAACC	Biomers, Ulm, Germany
GC binary oligomer	CCGGCCCGGCGGCCGGCGCG	Biomers, Ulm, Germany
UAG oligomer 1	AUU UUU UUU UUU UUU UUU UG	Biomers, Ulm, Germany
UAG oligomer 2	UUU UAU UUU UUU UUU GUU UU	Biomers, Ulm, Germany
UAG oligomer 3	UUU UUU UUA UUG UUU UUU UU	Biomers, Ulm, Germany
UAG oligomer 4	UUU UUU UUU AGU UUU UUU UU	Biomers, Ulm, Germany
oxo^8G 38mer	UCUGAGGGUCCAGGG-oxo^8G-UCAAGUCCCUGUUCGGGCGCCA	Bio-Synthesis Inc, Lewisville, USA
unmodified 38mer	UCUGAGGGUCCAGGGGUCAAGUCCCUGUUCGGGCGCCA	Biomers, Ulm, Germany
Abasic 38mer	UCUGAGGGUCCAGGG-X-UCAAGUCCCUGUUCGGGCGCCA X = abasic site	Synthesis described below
5.8S rRNA forward primer	TAATACGACTCACTATAGGAAACTTTCAACAACGGATCTCT TGG	Merck KGaA, Darmstadt, Germany
5.8S rRNA reverse primer	AAATGACGCTCAAACAGGCATG	Merck KGaA, Darmstadt, Germany
18S rRNA forward primer	TAATACGACTCACTATAGGTATCTGGTTGATCCTGCCAGTA G	Merck KGaA, Darmstadt, Germany
18S rRNA reverse primer	TAATGATCCTTCCGCAGGTTC	Merck KGaA, Darmstadt, Germany
25S rRNA -5' half forward primer	TAATACGACTCACTATAGGGTTTGACCTCAAATCAGGTAGG	Merck KGaA, Darmstadt, Germany
25S rRNA -5' half reverse primer	TTAACCGGATTCCCTTTCGATG	Merck KGaA, Darmstadt, Germany
25S rRNA -3' half forward primer	TAATACGACTCACTATAGGGATTCCGGAACCTGGATATGGA	Merck KGaA, Darmstadt, Germany
25S rRNA -3' half reverse primer	ACAAATCAGACAACAAAGGC	Merck KGaA, Darmstadt, Germany

Supplementary Methods

Polymerase Chain Reaction (PCR) of tRNA^{Asp}

tRNA^{Asp} was synthesized using a DNA template amplified by PCR. Reactions were carried out in a total volume of 200 µL including a final concentration of 16 mM $(NH_4)_2SO_4$ (Carl Roth, Germany), 67 mM Tris-HCl (Sigma-Aldrich, Germany) pH 8.8, 0.01% Tween 20 (Worthington Biochemical Corporation, USA), 3 mM $MgCl_2$ (Carl Roth, Germany), 400 µM dNTP mix (Thermo Fisher Scientific, Germany), 2 µM of each and 10 nM tRNA^{Asp} PCR template. Finally, 2.5 U *Taq* DNA polymerase (Thermo Fisher Scientific, Germany) was added. The reaction was initialized by denaturing for 2 min at 90°C followed by 35 cycles of annealing (30 s at 59°C), elongation (45 s at 72°C) and denaturation (30 s at 90°C). Final elongation was performed for 5 min before terminating the reaction by cooling to 4°C.

In vitro transcription of tRNA^{Asp}

400 µL of the PCR reaction product mixture was used without further purification for *in vitro* transcription (IVT) reactions. 1 mL IVT mixture was prepared including 5X T7 Transcription buffer (final concentration of 1X, Thermo Fisher Scientific, Germany), 30 mM $MgCl_2$ (Carl Roth, Germany), 5 mM DTT (Carl Roth, Germany), 5 mM of each NTP (Carl Roth, Germany), 2.5 µg/mL BSA (Thermo Fisher Scientific, Germany) and 120 U T7 polymerase (Thermo Fisher Scientific, Germany). Reactions were carried out at 37°C for 4 h. The DNA template was digested by adding 1 U DNase I, RNase-free (Thermo Fisher Scientific, Germany) and 100 µL 10X DNase I buffer (100 mM Tris-HCl, pH 7.5, 25 mM $MgCl_2$, 1 mM $CaCl_2$) to the transcription reaction followed by incubation for 1 h at 37°C and ethanol precipitation.

PCR of *S. cerevisiae* rRNA

S. cerevisiae IVT rRNA species were synthesized from PCR products obtained by amplification of pHW18 template. PCR reactions were carried out using 25 ng of pHW18 in a total volume of 50 µL, including 0.2 mM final dNTP mix (Thermo Fisher Scientific, Germany), 1X Pfu DNA Polymerase Buffer with $MgSO_4$ (Thermo Fisher Scientific, Germany), 0.5 µM of specific forward primers with T7 promoter and reverse primers for 18S, 5.8S and 25S rRNA (see Supplementary Table S5), and 1.25U Pfu DNA polymerase (Thermo Fisher Scientific, Germany). The reaction was initialized by a denaturation step for 5 min at 95°C followed by 15 cycles of denaturation (30 s at 95°C), annealing (30 s at 55°C) and elongation (30 s for 5.8S rRNA to 4 min for 18S and 25S rRNA templates, all at 72°C). A final elongation step at 72°C was performed for 5 min before storage at 4°C.

In vitro transcription of *S. cerevisiae* rRNA

50 µL of the PCR reaction product mixture was used without further purification for in vitro transcription (IVT) reactions. The transcriptions were carried out at 37°C for 6 h in the presence of 100 nM final PCR products, 60 U T7 RNA pol Plus (Thermo Fisher Scientific, Germany), 24 mM $MgCl_2$ (Thermo Fisher Scientific, Germany), 40 mM DTT (Thermo Fisher Scientific, Germany), 4 mM of each NTP (Thermo Fisher Scientific, Germany), 2 mM spermidine (Thermo Fisher Scientific, Germany), 80 mM HEPES-KOH pH 7.5 and 40 U RNase inhibitor RNasin® (Promega, Germany).

Phenol-based RNA extraction methods of *S. cerevisiae* IVT rRNA

S. cerevisiae in vitro-transcribed rRNA samples were subjected to either phenol-chloroform or acid phenol extraction. A mixture comprising 60% 25S, 30% 18S, and 10% 5.8S IVT *S. cerevisiae* rRNAs was prepared and subjected to these extraction methods. Concurrently, control samples were maintained without any additional RNA extraction. The entire experiment was conducted in duplicate, n = 2. Subsequently, 200 ng of the IVT rRNA samples were analyzed using AlkAnilineSeq protocol.

Ethanol precipitation

Ethanol precipitation was performed by adding 1 µL glycogen RNA grade (Thermo Fisher Scientific, Germany), 10 µL 5 M ammonium acetate (NH_4OAc, Sigma-Aldrich, Germany) and 300 µL ice-cold ethanol ≥ 96% (Carl Roth, Germany) to 100 µL sample. The mixture was vortexed and stored overnight at -20°C before centrifugation at 13,000 x*g* at 4°C for at least 45 min. The supernatant was removed and the pellet was washed with 70% ethanol followed by a second centrifugation step. Samples were air-dried and resolved in ultrapure water before determining the concentration on a Nanodrop™ One spectrophotometer by measuring the absorption at 260 and 280 nm.

Analytical denaturing polyacrylamide gel electrophoresis (PAGE)

RNA integrity was checked using PAGE. tRNA was analysed on a 10% (40% gel concentrate, 50% gel diluent and 10% gel buffer concentrate, all reagents Carl Roth, Germany) whereas oligomers were applied on a 15% urea gel (60% gel concentrate, 30% gel diluent and 10% gel buffer concentrate). 25 µL TEMED (Carl Roth, Germany) and 200 µL of a 10% (m/V) ammonium peroxydisulfate (APS, Carl Roth, Germany) solution were added to 50 mL solution, mixed and directly poured in the 1 mm interspace of two glass plates. Gels were run in 1X Rotiphorese™ TBE buffer (Carl Roth, Germany) for about 3 h at 18 W. 5 µL sample containing 100 ng RNA (250 ng for binary oligonucleotides stained with Toluidine blue) was loaded upon addition of 5 µL 2X denaturing loading buffer containing 10% 10X TBE, 90% formamide (Sigma-Aldrich, Germany). FastRuler Ultra Low Range DNA ladder (Thermo Fisher Scientific, Germany) was used according to manufacturer's instructions. Staining was performed using GelRed™ (Biotrend Chemikalien GmbH, Germany) followed by visualization a Typhoon™ TRIO+ imager (GE Healthcare) with following settings for GelRed™ (E_{Ex} = 532 nm, E_{Em} = 610 nm). Binary oligoribonucleotides were visualized using Toluidine blue (Thermo Fisher Scientific, Germany). Staining was performed in a 0.03% solution for 3 h followed by destaining the gel overnight in desalted water. Scans were performed on a commercial Epson Perfection V600 photo scanner (Epson Deutschland GmbH, Germany). ImageJ V5 (Version 1.52q) was used to edit gel scans and to create merged images. Displayed gels represent one out of three replicates, n = 3.

Preparative denaturing PAGE

In vitro transcribed RNA was purified by 10% denaturing PAGE after 3 h at 18 W (as described above). RNA was visualized by UV shadowing and the corresponding gel area was excised from the gel, gel pieces were mashed and transferred into 1.5 mL tubes. RNA was eluted in 0.5 M NH_4OAc before shaking overnight at 750 rpm and Nanosep filtering (0.45 µm, VWR, Germany) was performed according to the manufacturer's instructions. RNA was precipitated and dissolved in ultrapure water. The quality was checked on 10% denaturing PAGE gels after staining and visualizing as described above. The concentration was determined on a Nanodrop™ One spectrophotometer.

Quantification of free chlorine in hypochlorous acid (HOCl)

Calibration was performed using standard solutions including potassium iodate (KIO_3, Carl Roth, Germany) and potassium iodide (KI, Sigma-Aldrich, Germany) ranging from 0.5 mg/L until 5.0 mg/L KIO_3 in a final volume of 1000 µL. First, standards were filled up to 500 µL with ultrapure water and 10 µL of 1 M sulfuric acid (H_2SO_4, Carl Roth, Germany) was added, vortexed, spun down and incubated for 1 min to oxidize KIO_3. The reaction was quenched by adding 10 µL 2 M sodium hydroxide solution (NaOH, VWR, Germany) followed by the addition of 480 µL ultrapure water to fill up to 1000 µL. 50 µL of a DPD buffer pH 6.5 (46 g/L KH_2PO_4, 60.5 g/L Na_2HPO_4 * 12 H_2O, 0.8 g/L EDTA * 2 H_2O, all Carl Roth, Germany) and 50 µL of a freshly prepared DPD solution (1.1 mg/mL DPD, 0.2% H_2SO_4, 0.2 g/L EDTA * 2 H_2O) were added. To determine the concentration of free chlorine in HOCl (Thermo Fisher Scientific, Germany), 1 mL of a 1/5000 dilution in ultrapure water was prepared and the DPD buffer and DPD solution were added. The slope of the resulting calibration curve was used for quantification purposes, with KI_3 equivalents being converted into Cl_2 amounts according to the conversion factor of 10.06 µg KI_3 = 0.141 µmol Cl_2. Cl_2 quantification was conducted every two weeks. The influence of different pH values (6.4, 7.4, 8.4), EDTA concentrations and potential quenching agents were investigated according to the following adaptations: 50 mM phosphate buffer and 10 mM potential quenching agents (methionine, Sigma-Aldrich, Germany; Tris buffer pH 6.7, Carl Roth, Germany; ammonium bicarbonate, Sigma-Aldrich, Germany); dithiothreitol, Carl Roth, Germany; sodium thiosulfate, Sigma-Aldrich, Germany; potassium bicarbonate,

Sigma-Aldrich, Germany; ammonium acetate, Sigma-Aldrich, Germany; β-mercaptoethanol, Sigma-Aldrich, Germany; ammonium thiosulfate, Sigma-Aldrich, Germany) were added to the HOCl solution before DPD buffer and DPD solution addition. The influence of EDTA was analyzed by adapting the EDTA concentration in the DPD buffer and DPD solution to final concentrations of 0.0, 0.2, 0.4 and 0.8 g/L. The Cl_2 content was considered as constant for two weeks and used for further calculations. Experimental replicates were performed with n = 3 in Figure **S2 B, C** and n = 9 for unbuffered conditions and n = 2 for other components analyzed in **D**.

pH analysis of oxidation reactions

Samples were prepared according to the standard oxidation conditions for analyzing the pH value of an oxidation series. Samples with a final volume of 1000 µL were prepared with Cl_2 concentrations of 0, 10, 25, 50, 75, 100, 250 and 500 µM in 50 mM potassium phosphate buffer (Carl Roth, Germany) at pH 7.40 containing 5 mM $MgCl_2$ (Thermo Fisher Scientific, Germany). The pH was measured using a pH electrode (LE422, Mettler Toledo, Germany) at the start of the experiment (0 minutes) and again after 10 minutes. To analyze the influence of RNA, an *in vitro*-transcribed RNA transcript was added to a final concentration of 50 ng/µL. Each condition was tested three times (n = 3).

Nucleoside oxidation

Oxidation reactions were carried out using final concentrations of 100 µM nucleoside (Sigma-Aldrich, Germany) in 50 mM phosphate buffer (potassium salt) pH 7.4 (Carl Roth, Germany) and 5 mM $MgCl_2$ (Carl Roth, Germany) in a total volume of 120 µL. Free Cl_2 in HOCl (Thermo Fisher Scientific, Germany) was quantified before oxidation experiments. Concentrations of Cl_2 ranged from 0 µM to 250 µM Cl_2. 60 µL of the phosphate-buffered stock solution (200 µM nucleoside, 10 mM $MgCl_2$) was prepared and 60 µL of phosphate-buffered HOCl solutions (2X of final concentrations) was added, the mixture was vortexed, spun down and incubated for 10 min at 25°C in the dark. Quenching was performed by adding DTT (Carl Roth, Germany) to a final concentration of 10 mM, samples were vortexed and stored at -20°C.

Synthetic oligomer and tRNA oxidation

Oxidation reactions were carried out using final concentrations of 25 ng/µL RNA in 50 mM phosphate buffer (potassium salt) pH 7.4 and 5 mM $MgCl_2$ in a total volume of 120 µL with concentrations of free chlorine ranging from 0 µM to 1600 µM. Therefore, 60 µL of phosphate-buffered stock solution including 50 ng/µL RNA and 10 mM $MgCl_2$ was prepared in Eppendorf tubes. HOCl was diluted resulting in 2X of final concentrations and 60 µL was added to the prepared RNA-containing tube, the mixture was vortexed, spun down and incubated for 10 min at 25°C. Quenching was performed by adding DTT (Carl Roth, Germany) to a final concentration of 10 mM, samples were vortexed and directly put on ice. To check RNA integrity, an aliquot equal to 100 ng of initially present RNA was taken for PAGE analysis. The reaction mixture was purified by ethanol precipitation, filtering through 0.2 µm Nanosep centrifugal filters (Pall corporation, Germany), buffer exchange using Zeba™ Spin Desalting Columns (Thermo Fisher Scientific, Germany) according to manufacturer's instructions followed by phenol/chloroform extraction (Carl Roth/Sigma-Aldrich, Germany) and a second EtOH precipitation. RNA concentration was determined on a NanoDrop™ One spectrophotometer. Experiments were performed in triplicates.

In vitro oxidation of "naked" *S. cerevisiae* RNA

Yeast cells (BY4742α) were grown in standard Yeast Extract/Peptone/Dextrose (YPD) media at 30°C with shaking at 180 rpm until the mid-exponential growth phase (0.6 – 0.8 OD_{600}). Cells were harvested and total RNA was isolated using hot acid phenol technique. The RNA concentration was measured on a Nanodrop™ One and RNA quality was assessed by capillary electrophoresis using a Pico RNA chip on Bioanalyzer 2100 (Agilent Technologies, USA). For oxidation reaction, 25 ng/µL of extracted *S. cerevisiae* RNA samples were subjected to HOCl in concentrations of free chlorine ranging from 10- 500 µM with 50 mM phosphate buffer (with potassium salt), pH 7.4 and 5 mM $MgCl_2$ in a total volume of 120 µL. The non-oxidized (0 µM) controls were incubated with similar buffer conditions, except HOCl was replaced with an equivalent volume of RNase-free water. As HOCl is highly reactive, the 60 µL reaction mix without HOCl was prepared in Eppendorf tubes in advance (50 ng/µL RNA in 50 mM phosphate buffer and 10 mM $MgCl_2$). To

this mixture, 60 µL of a 2X concentration of free chlorine were then added. The reaction was mixed by vortexing, spin down and incubated for 10 min at 25°C. Quenching was performed by adding DTT to a final concentration of 10 mM in treated and control samples, samples were vortexed and directly put on ice. The oxidized RNA and control samples were purified from the reaction components by ethanol precipitation, buffer exchange using Zeba™ Spin Desalting Columns (Thermo Fisher Scientific, Germany) according to manufacturer's instructions followed by phenol/chloroform extraction and a second EtOH precipitation. The experiment was performed in biological triplicates. Following the oxidative treatment, a reassessment of both the quantity and quality of the oxidatively damaged RNA was conducted, as previously described.

In vitro oxidation of *S. cerevisiae* ribosomes (RNP)

BY4742α yeast cells were grown at 30°C in YPD medium. Whole cell extracts were prepared from exponentially growing cultures as described in Pospisek and Valasek, 2013[1]. In brief, at an OD_{600} of 1.0, 0.18 µM (50 µg/mL) of cycloheximide was introduced to the culture 5 min before harvesting. The cells were pelleted at 3000 x*g* for 5 min at 4°C using pre-cooled polypropylene tubes. The pellets were reconstituted in buffer A (at a volume 1.3 times that of the pellet weight) comprising 20 mM Tris-HCl (pH 7.4), 50 mM KCl, 10 mM $MgCl_2$, and 1 mM DTT. Equal volumes of glass beads were added and cells were lysed by repeated cycles (8 times) of vigorous vortexing (30 s) followed by 1 min on ice. Lysed cells are then centrifuged at 3000 x*g* for 5 min at 4°C. The resulting supernatant containing the whole cell extract (WCE) was collected. To ensure purity, the WCE underwent two rounds of centrifugation at 11300 x*g* for 2 min and 10 min, both at 4°C. Subsequently, ribosomal complexes were separated by sucrose density ultracentrifugation. Linear sucrose gradients (5% – 40%) were prepared in whole cell extract. Separation was achieved by centrifuging the samples in a SW40 Ti rotor for 2.5 h at 272076 x*g* at 4°C. The gradients were then fractionated using the Density Gradient Fractionation system, with continuous scanning at 254 nm, and the collected fractions were subjected to analysis. Ribosomes were isolated from monosomal (F2) and polysomal (F3) fractions, initially in Buffer A containing DTT. To eliminate DTT, the isolated ribosomes underwent buffer exchange using PD MiniTrap G-25 columns, transitioning to a 50 mM phosphate buffer pH 7.4, suitable for subsequent HOCl treatment. Ribosomes, corresponding to 1000 ng of RNA, were then incubated in a phosphate buffer with 5 mM $MgCl_2$, along with HOCl in a final volume of 120 µL. The reactions spanned concentrations of free chlorine ranging from 0 µM to 100 µM. The non-oxidized (0 µM Cl_2) controls were incubated with similar buffer conditions, except that HOCl was replaced with an equivalent volume of RNase-free water. Subsequently, the oxidation reaction was quenched using 10 mM DTT and RNA was extracted from the ribosomes using TRIzol™ reagent (Thermo Fisher Scientific, Germany) following the manufacturer's instructions. The control samples underwent similar quenching and RNA isolation procedures. Finally, the RNA quality and quantity were assessed as described previously.

In vivo oxidation of *S. cerevisiae* cells

S. c. cells (BY4742α) were grown in standard Yeast Extract/Peptone/Dextrose (YPD) until the mid-exponential phase (0.71 OD_{600}). The cultured cells were subjected to rigorous washing procedures to remove any traces of media. Firstly, they were washed twice with RNase-free water followed by three washes with 1X Phosphate-Buffered Saline (PBS). Thereafter, the cells were resuspended in 1X PBS. The HOCl oxidation treatment was performed using increasing concentrations ranging from 0 mM to 20 mM Cl_2. The non-oxidized (0 mM Cl_2) controls were incubated with similar buffer conditions, except HOCl was replaced with an equivalent volume of RNase-free water to a final volume of 400 µL. The cells were exposed to HOCl for 10 min at 25°C in a thermomixer spinning at 350 rpm. Following the oxidative treatment, the reaction was promptly quenched by the addition of 20 mM DTT. The total RNA was isolated using hot acid phenol. The control samples underwent similar quenching and RNA isolation procedures. Finally, the RNA quality and quantity were assessed as described previously.

PolyA enrichment of *S. cerevisiae* mRNA from *in vivo* RNA oxidized samples

mRNA was isolated from total *S. cerevisiae* RNA using the NEBNext® Poly(A) mRNA Magnetic Isolation Module (New England Biolabs GmbH, Germany). Total RNA was extracted from *in vivo* oxidized yeast cells as described above. The volume of 5 µg total RNA was adjusted to 50 µL with RNAse-free water. Oligo d(T)25 beads were prepared according

to the manufacturer's protocol. Total RNA was added to the Oligo d(T)25 beads suspension, mixed thoroughly, heated for 5 min at 65°C and rapidly cooled down to 4°C. The mixture was mixed slowly 10 times and incubated for 5 minutes at RT and placed on the magnet until the solution was clear. The supernatant was discarded and the mRNA-bead complex was washed twice with 200 µL Wash Buffer. Afterwards, a second round of binding was performed to recover higher yields of mRNA. mRNA was then eluted at 80°C for 2 min followed by a cool down at 25°C and placed immediately on the magnet. The eluted RNA was transferred into a new tube before being subjected to AAS.

RNA extraction from mouse tissue

All experiments were carried out in accordance with the National Institutes of Health guidelines for the humane treatment of animals and the European Communities Council Directive (86/609/EEC). The C57BL/6J mice used in this study were treated as described in Kopietz, Raorane *et al.*, 2025[2]. Mice were housed under a 12h dark/light cycle, temperature and humidity were kept constant (~ 20°C, ~ 50%). First, they were anesthetized with CO_2 and then euthanized by cervical dislocation. At the time of sacrifice, multiple tissues were dissected, including regions of the brain (cortex, cerebellum, hippocampus). These tissues were transferred into cryotubes containing RNAlater (Thermo Fisher Scientific, USA) and stored at 4°C for 24 hours to allow them to harden. After this period, excess RNAlater was removed, and the samples were stored at -80°C until RNA extraction. Before RNA isolation, frozen tissues were allowed to thaw at RT and then kept on ice. Any remaining RNAlater was removed and the tissue samples were weighed and transferred into polypropylene tubes and kept on ice. Each sample was mixed with 1 mL TRIzol reagent (Invitrogen, Thermo Fisher Scientific, USA) and homogenized using an Omni TH homogenizer at medium speed for 15-30 seconds. The homogenates were transferred into microcentrifuge tubes and kept at RT. Chloroform was added to each homogenate followed by brief vortexing and incubation at 15-30°C for 2-3 minutes. Samples were centrifuged at maximum speed at 4°C for 15 minutes. The upper phase was carefully transferred to fresh tubes, mixed with an equal volume of isopropanol, vortexed for 5 seconds and left to incubate at RT for 2-3 minutes. A second centrifugation was performed at 12,000 *xg* for 10 minutes at 4°C. After discarding the supernatant, RNA pellets were washed with 500 µL of 75% ethanol, partially resuspended by vortexing and centrifuged again for 2-3 minutes at RT. The ethanol was removed, the pellet was air-dried before being dissolved in 25-50 µL RNAse-free water.

LC-MS/MS measurement of oxygenated and chlorinated nucleosides

The Triple Quadrupole mass spectrometer was supplied with an electrospray ion source (Agilent Jet Stream) and run at a gas temperature of 300°C, gas flow 7 L/min, nebulizer pressure 60 psi, sheath gas temperature 400°C, sheath gas flow 12 L/min and capillary voltage 3000 V. The following parameters were used for the detection of chlorinated and oxidized nucleosides:

Supplementary Table 6

Compound	Precursor Ion	Product Ion	Fragmentor voltage [V]	Collision energy [V]	Cell accelerator voltage [V]	Retention Time [min]	Delta Retention Time [min]
ho^5C	260	128	80	9	4	4.4	4
ho^5U	261	129	80	9	4	5.2	4
Cl^5C	278	146	75	13	4	15.5	4
Cl^5U	279	147	75	13	4	17.2	4
oxo^8A	284	152	90	21	4	24.4	4
oxo^8G	300	168	100	13	4	18	4
Cl^8A	302	170	95	17	4	33.3	4
Cl^8G	318	186	85	17	4	27	4

The four main nucleosides detected and quantified via UV eluted at 4.9 min (cytidine), 6.8 min (uridine), 15.7 min (guanosine) and 23.5 min (adenosine).

LC-MS/MS measurement of 5'-ribose phosphate and NMPs

The Triple Quadrupole mass spectrometer was supplied with an electrospray ion source (Agilent Jet Stream) run at a gas temperature of 300°C, gas flow 7 L/min, nebulizer pressure 60 psi, sheath gas temperature 400°C, sheath gas flow 12 L/min and capillary voltage 3000 V. Following parameters were used for the detection of the 5'-ribose phosphate and oxo^{8}GMP:

Supplementary Table 7

Compound	Precursor Ion	Product Ion	Fragmentor voltage [V]	Collision energy [V]	Cell accelerator voltage [V]	Retention Time [min]	Delta Retention Time
5'-RP	231	97	85	31	5	1.9	5
oxo^{8}GMP	380	168	100	13	5	3.4	5

The four main nucleotides eluted at 2.3 min (CMP), 2.8 min (UMP), 3.4 min (GMP) and 8.3 min (AMP).

LC-MS measurements of ^{13}C- and ^{15}N- labelled guanosine

Neutral loss scans were used to identify alternative Guanosine oxidation products, where no synthetic standards were commercially available. In order to detect oxidized nucleosides shown in Figure S5, a 100 µM solution of ^{13}C- and ^{15}N-isotope labelled G (Silantes, Germany) was oxidized using 50 µM Cl_2 according to the procedure described above (nucleoside oxidation). 10 pmol nucleoside was injected into the LC-MS system and the loss of 132 Da and 137 Da was detected in a scan range for *m/z* = 230 to 600. ^{12}C- and ^{15}N- nucleosides are detected by the loss of 132 Da, whereas the mass of the ^{13}C-labeled nucleoside is reduced by 137 Da. Additional method settings were as follows: fragmentor voltage 80 V, collision energy 0 V, cell accelerator voltage 5 V, scan time 500 ms. Resulting mass transitions are depicted in Supplementary Table S3. As no external standards were available for the open-ring oxidation products, the MS signals were only normalized to the total of present nucleosides in untreated control samples.

Abasic site labeling

Abasic site labeling was performed using an aldehyde reactive probe (ARP) reagent: Alexa Fluor™ 488 hydroxylamine (Thermo Fisher Scientific, Germany) which selectively reacts with the open-ring aldehyde form of the ribose moiety at abasic sites. The reaction was conducted in a total volume of 10 µL including 20 mM HEPES pH 7.2 (Carl Roth, Germany), 2 mM EDTA (Carl Roth, Germany), 600 ng of oxidized RNA and 100 µM ARP reagent (Alexa Fluor™ 488 hydroxylamine, Thermo Fisher Scientific, Germany). Incubation was performed for 2 h at 37°C in the dark followed by addition of 40 µL ultrapure water to terminate the reaction. To isolate labeled oligomers from excess ARP reagent, the Oligo Clean & Concentrator kit (Zymo Research Europe GmbH, Germany) was used. Absorbance spectra were recorded on a Nanodrop™ One device in a range of 220 nm to 850 nm. Analysis of labeling reactions were performed via analytical 15%-denaturing PAGE and LC-MS in a dynamic MRM mode (described above). Scans were performed on a Typhoon TRIO+ imager (GE Healthcare) with following settings: GelRed™ (E_{Ex} = 532 nm, E_{Em} = 610 nm), AlexaFluor™ 488 (E_{Ex} = 488 nm, E_{Em} = 526 nm). ImageJ was used to edit gel scans and to create merged images.

Synthesis of 1'-[(*R*,*S*)-1-(2-nitrophenyl)ethyl]-5'-*O*-(4,4'-dimethoxytrityl)- 2'-*O*-[(1,1-dimethylethyl)dimethylsilyl]-3'-*O*-(2- cyanoethyl-*N*,*N*-diisopropyl) phosphoramidite

Synthetic procedures are based on Küpfer, Leumann *et al.* 2007[3].

1'- [(*R*,*S*) -1-(2-nitrophenyl) ethyl] - 2',3',5'-tri-*O*-benzoyl-ribofuranose (I)

1-*O*-Acetyl-2,3,4-tri-*O*-benzoyl-*β*-*D*-ribofuranose (2.35 g, 4.67 mmol, 1.20 eq.) was dissolved in 20 mL dry acetonitrile under argon. The solution was cooled to -20°C and 1-(2-nitrophenyl) ethanol (515 µL, 3.89 mmol, 1.00 eq.) were added. Trimethylsilyl trifluoromethanesulfonate (246 µL, 1.36 mmol, 0.35 eq.) was added in four portions over two hours. After

another 2 h of stirring, the solution was poured into 50 mL of ethyl acetate and washed three times with saturated sodium bicarbonate solution. The combined organic phases were dried with anhydrous sodium sulfate and filtered. The solvent was removed on the rotary evaporator and the crude compound purified via column chromatography (SiO_2, ethyl acetate/ n-hexane: 1/9 → 4/6) to give a yellow oil (diastereomeric mixture).

Yield: 2.22 g (3.63 mmol, 93%)

TLC (*n*-hexane/ ethyl acetate: 3/1): R_f = 0.30

1H-NMR (400 MHz, $CDCl_3$, 25°C): δ 8.10-7.30 (m, 38H), 5.89 (t, 1H), 5.75 (t, 1H); 5.56-5.47 (2m, 2H), 5.77-5.70 (m, 2H), 5.44 (s, 1H), 5.03 (s, 1H), 4.77-4.57 (2m, 4H), 4.41-4.05 (2m, 2H), 1.58 (d, 3H), 1.55 (d, 3H).

1'-[(*R*,*S*)-1-(2-nitrophenyl)ethyl]-ribofuranose (II)

To a solution of compound **I (**2.22 g, 3.63 mmol, 1.00 eq.) in methanol (20 mL) sodium carbonate (385 mg, 3.63 mmol, 1.00 eq.) was added under argon. The mixture was stirred for 20 hours at room temperature. Ethyl acetate (150 mL) was added to the reaction mixture and the resulting solution was washed four times with saturated sodium bicarbonate solution. The organic phase was dried over anhydrous sodium sulfate, filtered, and the solvent was evaporated under reduced pressure. The product was purified by column chromatography (SiO_2, ethyl acetate) to give compound **II** as a white solid (diastereomeric mixture).

Yield: 0.771 g (2.58 mmol, 71%)

TLC (ethyl acetate): R_f = 0.37

1H-NMR (400 MHz, DMSO-d_6, 25°C): δ 7.91 (m, 2H), 7.73 (m, 4H), 7.52 (m, 2H), 5.19-5.11 (m, 4H), 5.03 (m, 1H), 4.94 (m, 1H), 4.89 (d, 1H), 4.78 (m, 2H), 4.67 (m, 1H), 4.41 (d, 1H), 4.34 (m, 1H), 3.90 (m, 1H), 3.77-3.63 (m, 5H), 3.48 (m, 2H), 3.12 (m, 2H), 1.41 (2d, 6H).

1'-[(*R*,*S*)-1-(2-nitrophenyl)ethyl]-2'-*O*-(*tert.*-butyldimethylsilyl)-3',5'-*O*-(di-tert-butyl) silyl-ribofuranose (III)

Compound **II** (766 mg, 2.56 mmol, 1.00 eq.) was dissolved in dry dimethylformamide (15 mL) under argon. Di-tert-butylsilyl ditriflate (917 µL, 2.82 mmol, 1.10 eq.) was added slowly at 0°C and the solution was stirred for 60 min. After removal of the ice bath imidazole (871 mg, 12.8 mmol, 5.00 eq.) was added and the mixture was stirred for 30 min at room temperature. Then tert-butyl dimethyl silyl chloride (502 mg, 3.33 mmol, 1.30 eq.) was added and stirred at 60°C for another 90 min. Dimethylformamide was then evaporated under reduced pressure and the oily residue dissolved in chloroform. The solution was washed with saturated sodium chloride solution three times. The organic phase was dried over anhydrous sodium sulphate and filtered. Subsequently, chloroform was removed under reduced pressure and the crude product was purified by column chromatography (SiO_2, n-hexane/ethyl acetate: 9/1). Compound **III** was obtained as a white foam (diastereomeric mixture).

Yield: 1.09 g (1.97 mmol, 77%)

TLC: (*n*-hexane/ethyl acetate: 9/1): R_f = 0.54

1H-NMR (400 MHz, $CDCl_3$, 25°C): δ 7.89 (m, 2H), 7.63 (m, 4H), 7.41 (m, 2H), 5.36-5.20 (2m, 2H), 4.93 (m, 1H), 4.54 (m, 1H), 4.38 (m, 1H), 4.12-4.00 (m, 5H), 3.97-3.83 (m, 3H), 3.21 (m, 1H), 1.52 (2d, 6H), 1.07-0.81 (6s, 54H), 0.13-0.024 (s, 2H).

1'-[(*R*,*S*)-1-(2-nitrophenyl)ethyl]-2'-*O*-(*tert.*-butyldimethylsilyl)-ribofuranose (IV)

Compound **III** (1.09 g, 1.97 mmol, 1.00 eq.) was dissolved in dry dichloromethane (12 mL) under argon. After the addition of a mixture of pyridine (1.23 mL, 15.3 mmol, 7.75 eq.) and hydrogen fluoride (197 µL, 70%, 7.58 mmol, 3.85 eq.), the reaction mixture was stirred for 90 min at 0°C under argon. The solution was diluted with dichloromethane and

washed three times with saturated sodium bicarbonate solution. The combined organic phases were dried over anhydrous sodium sulfate. After filtration the solvent was evaporated under reduced pressure. The crude product was used without further purification for the next step.

Yield: quant.

TLC: (ethyl acetate/*n*-hexane: 1/1): R_{f1} = 0.35, R_{f2} = 0.43

1'-[(*R*,*S*)-1-(2-nitrophenyl)ethyl]-5'-*O*-(4,4'-dimethoxytrityl)-2'-*O*-(*tert.*-butyldimethylsilyl)-ribofuranose (V)

Compound **IV (**814 mg, 1.97 mmol, 1.00 eq.) was co-evaporated three times with pyridine and then redissolved in dry pyridine (9 mL) under argon. 4,4′-dimethoxytrityl chloride (734 mg, 2.17 mmol, 1.10 eq.) was added in three portions and the mixture stirred for 90 min at room temperature under argon. The reaction was quenched with methanol. The solvent was evaporated and the oily residue diluted with dichloromethane. The solution was washed five times with a 5% citric acid solution. The organic phase was washed again three times with saturated sodium bicarbonate solution. The organic phase was dried over anhydrous sodium sulfate and the solvent in the filtrate was evaporated under reduced pressure. After the purification via column chromatography (SiO_2, n-hexane/ethyl acetate: 7/3), compound **V** was obtained as a slightly yellowish foam (diastereomeric mixture).

Yield: 1.24 g (1.73 mmol, 88%)

TLC: *n*-hexane/ethyl acetate: 7/3): R_{f1} = 0.49, R_{f2} = 0.54

1H-NMR (400 MHz, $CDCl_3$, 25°C): δ 7.91 (m, 2H), 7.65 (m, 4H), 7.48 (m, 2H), 7.42-7.16 (m, 18H), 6.87-6.74 (m, 8H), 5.48-5.37 (2m, 2H), 5.05 (d, 1H), 4.30-3.92 (m, 4H), 3.80 (s, 6H), 3.78 (s, 6H), 3.36 (m, 1H), 2.93 (m, 1H), 2.54 (m, 2H), 2.33 (m, 2H), 1.53 (d, 3H), 1.43 (d, 3H), 0.94 (s, 9H), 0.85 (s, 9H), 0.18-(-0.02) (4s, 12H).

1'-[(*R*,*S*)-1-(2-nitrophenyl)ethyl]-5'-*O*-(4,4'-dimethoxytrityl)-2'-*O*-(*tert.*-butyldimethylsilyl)-3'-*O*-(2-cyanoethyl-*N*,*N*-diisopropyl) phosphoramidite (VI)

Compound **V (**1.24 g, 1.73 mmol, 1.00 eq.) was dissolved in dry acetonitrile (7 mL) under argon. After the addition of 5-(benzylthio)-1H-tetrazole (7.60 mL, 1.90 mmol, 1.10 eq.), the solution was stirred at room temperature for 30 min. Then 2-cyanoethyl-*N,N,N',N'*-tetraisopropyl- phosphorodiamidite (1.10 mL, 3.45 mmol, 2.00 eq.) was added and the reaction mixture was stirred for overnight at room temperature under argon. The solution was then washed two times with half-saturated sodium bicarbonate solution. The organic phase was dried over anhydrous sodium sulfate, the solvent in the filtrate was evaporated under reduced pressure and column chromatography (SiO_2, n-hexane/ethyl acetate 7/3) was performed to obtain the pure product **8** as a slightly yellowish foam (diastereomeric mixture).

Yield: 1.15 g (1.73 mmol, 73%)

TLC: *n*-hexane/ethyl acetate: 7/3): R_{f1} = 0.56, R_{f2} = 0.61

1H-NMR (400 MHz, $CDCl_3$, 25°C): δ 7.95 (m, 2H), 7.72 (m, 4H), 7.55 (m, 2H), 7.45-7.15 (m, 18H), 6.88-6.74 (m, 8H), 5.55-5.40 (m, 2H), 5.04 (d, 1H), 5.02 (d, 1H), 4.35-4.04 (m, 4H), 3.80 (s, 6H), 3.78 (s, 6H), 3.74-3.33 (m, 8H), 3.22 (m, 1H), 2.87 (m, 1H), 2.59 (m, 2H), 2.30 (m, 2H), 1.54 (d, 3H), 1.45 (d, 3H), 1.13-1.07 (m, 24H), 0.93 (s, 9H), 0.81 (s, 9H), 0.15-(-0.07) (4s, 12H). 31P-NMR (162 MHz, CDCl3, 25°C): δ 149.57 (s), 149.00 (s), 148.76 (s), 148.38 (s). ESI-MS (*m/z*) calculated for $C_{49}H_{66}N_3O_{10}PSi$: $[M+H]^+$ = 916.43, found $[M+H]^+$ = 916.43, $[M+Na]^+$ = 938.41,

found $[M+Na]^+$ = 938.41.

Chemical synthesis of abasic site modified RNAs

Standard 2'-*O*-TBDMS RNA phosphoramidites (rA^{Ac}, rC^{Ac}, rG^{Ac} and rU, *Chemgenes*, USA) were used in combination with in-house synthesized RNA 1'-[(*R*,*S*)-1-(2-nitrophenyl)ethyl]-abasic phosphoramidite. Controlled pore glass (CPG)

RNA solid support (1000 Å pore size, *ChemGenes*, USA) with an average loading of 40 µmol g^{-1} was used to synthesize the RNAs on an ABI 391 PCR Mate using a self-written RNA synthesis cycle. Amidite (0.1 M) and activator (5-benzylthio-*1H*-tetrazole, 0.25 M) solutions were dried over freshly activated molecular sieves (3 Å) for at least 48 hours. The following reagent mixtures were used: *Cap A*: acetic anhydride/lutidine/tetrahydrofuran 1/1/8, v/v/v. *Cap B*: tetrahydrofuran/N-methylimidazole 86/16 v/v. *Oxidation solution*: 500 mg iodine dissolved in a mixture of 70 mL THF, 20 mL pyridine and 10 mL water. *Detritylation solution*: 4% dichloroacetic acid in anhydrous toluene. After complete RNA synthesis, the solid support was dried for 1 h in a high vacuum. *Mild alkaline deprotection*: 1 mL methanol and 1 mL ammonia (7 N) in methanol were added to the solid support. The reaction tube was shaken vigorously and incubated at room temperature for 48 h. The solid support was pelleted via centrifugation and the supernatant was transferred to a 10 mL round bottom flask. The remaining solid support was washed three times with a mixture of THF/water (1/1), the liquid phases were combined with the first filtrate and evaporated to dryness. The residual white precipitate was dried in high vacuum for at least 1 h. *2'-O-TBDMS deprotection:* The residue from the previous step was dissolved in 300 µL anhydrous dimethyl sulfoxide, and triethylamine trihydrofluoride was added (50 eq. per TBDMS group) and the deprotection mixture incubated at 37°C for at least 16 h. Then, the deprotection mixture was quenched with 2 mL quenching buffer (*GlenResearch*, USA) and directly applied to a HiPrep 26/10 desalting column (*GE Healthcare*, Austria) using a ÄKTA start system (*GE Healthcare*, Austria). The crude RNA was eluted using HPLC grade water and the RNA containing fractions (UV detection at 254 nm) were collected in a 50 mL round bottom flask. After evaporation, the crude RNA was dissolved in 1 mL HPLC grade water and transferred to a 1.5 mL reaction tube. The crude RNA was stored at -20°C. The quality of the crude RNAs was checked via anion exchange chromatography on an analytical Dionex DNAPac PA-200 column (4x250 mm; *Eluent A*: 25 mM Tris-HCl, 10 mM sodium perchlorate, 20 % acetonitrile, pH 8.0; *Eluent B*: 25 mM Tris-HCl, 600 mM sodium perchlorate, 20% acetonitrile, pH 8.0) and at elevated temperature (80°C). Purification of the RNA sequences was achieved in a single run by applying the crude RNA to a preparative Dionex DNAPac PA-200 column (22x250 mm, eluents as before). The fractions containing the desired RNA were pooled and loaded on a C18 SepPak cartridge (*Waters*, Austria) to remove HPLC buffer salts. The RNA sodium salt form was then eluted from the C18 column with water/acetonitrile (1/1, v/v), concentrated and transferred to a 1.5 mL reaction tube for concentration determination and mass spectrometric analysis. Sample concentrations were determined by measuring UV absorption at 260 nm on a NanoPhotometer (*Implen*).

The removal of the 1'-[(*R*,*S*)-1-(2-nitrophenyl)ethyl protecting group to liberate the abasic site was performed by irradiation of the dissolved RNA in a quartz cell with a UV-handlamp at 365 nm (6 Watt tube) for 30 min. Confirmation of complete removal was indicated via anion exchange chromatography of the RNA.

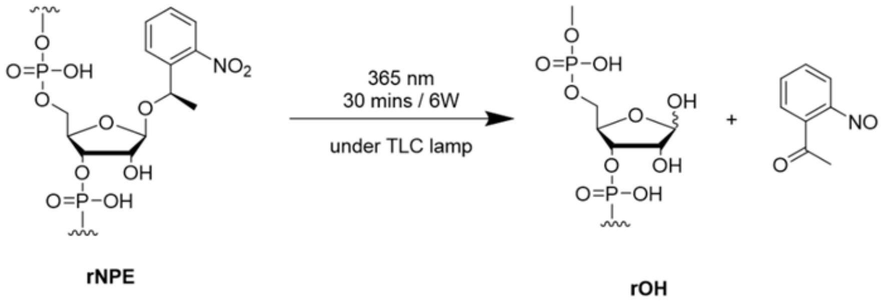

Supporting Scheme 1. Reaction scheme for the removal of the 1'-[(*R*,*S*)-1-(2-nitrophenyl)ethyl protecting group to generate an RNA abasic site.

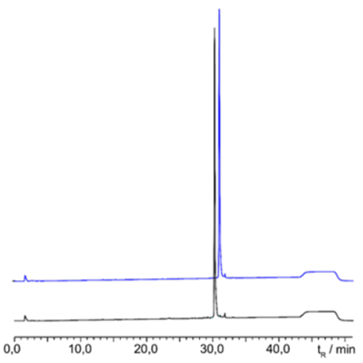

Supporting Figure 11. Overlay of anion exchange chromatograms of the abasic site containing RNA before (blue) and after irradiation (black) with 365 nm UV light for 30 minutes.

LC-ESI mass spectrometry

The abasic site containing RNA was analyzed on Finnigan LCQ Advantage MAX ion trap instrumentation connected to a Thermo Scientific UHPLC (components: Ultimate 3000 RS Pump, Ultimate 3000 RS Autosampler, Ultimate 3000 RS Column Compartment, Ultimate 3000 Diode Array Detector). RNA mass spectra were acquired in the negative-ion mode with a potential of -4 kV applied to the spray needle (capillary temperature: 270°C, capillary voltage: - 23V). LC: 250 pmol RNA dissolved in 30 µL of 20 mM ethylenediaminetetraacetic acid (EDTA) solution; average injection volume: 30 µL; column: Waters xBridge C18 2.5 µm column (1.0 × 50 mm) at 30°C; flow rate: 100 µL/min; Eluent A: 8.6 mM triethylamine (TEA), 100 mM 1,1,1,3,3,3-hexafluoroisopropanol in H_2O (pH 8.0); Eluent B: methanol; gradient: 0–100% B in A within 30 min; UV detection at 260/280 nm. The correct assembly of the RNA used in this study was confirmed by the mass data. LC-ESI-MS spectra were recorded with an intact 1'-[(*R*,*S*)-1-(2-nitrophenyl) ethyl protecting group for the abasic site.

MS (calculated) = 12239.32 g/mol
MS (found) = 12238.88 g/mol
Δmolecular weight = 0.44 g

Detecting oxo^8G and Abasic Sites in RNA mixture using AAS and OAb

To determine the signal strength of AAS and OAbSeq, unmodified 38mer, oxo^8G 38mer and abasic 38mer were mixed in varying proportions (as shown below in the table), diluted to a total volume of 20 µL, and then equally divided into two PCR tubes. To each tube, either 10 µL of bicarbonate buffer or 10 µL of Mg^{2+} was added to the tubes and AAS and OAbSeq were performed as described above.

Supplementary Table 7

oxo^8G 38mer [%]	abasic 38mer [%]	unmodified 38mer [%]
0	0	100
0	1	99
0	2	98
0	5	95
2.5	0	97.5
5	0	95
10	0	90
25	0	75
50	0	50
50	1	49
50	2	48
50	5	45
75	0	25
75	1	24
75	2	23
75	5	20
95	1	4
95	2	3
95	5	0
100	0	0

AAS and data analysis of mouse miRNAs, polyA-enriched *S. cerevisiae* mRNA and SCR1 RNA

AAS analysis of mouse miRNAs was performed using total RNA preparations from mouse brain tissues described above. The same AAS protocol was used, the resulting libraries were sequenced at 15-20 millions of raw reads depth. To avoid reads' mismapping to highly abundant RNA species, mouse rRNA and tRNA reads were filtered out and the remaining non-aligned reads were re-aligned to the mouse miRBase v22.1[4,5,6,7,8,9]. Bioinformatic analysis was performed in the same way as described above for rRNAs. AAS of *S. cerevisiae* mRNAs was conducted on enriched polyA+ fraction, extracted and enriched as described above. Sequencing depth for samples was ~15 millions of raw reads which allowed for coverage of a few highly abundant mRNA species. Alignment was done to the non-overlapping mRNA transcriptome reference derived from S288c_R64 *S. cerevisiae* genome assembly (Saccharomyces_cerevisiae - Ensembl Genomes 61). Further extraction of AAS scores and analysis was done as for rRNA species. AAS reads covering SCR1 (SRP RNA) were extracted directly from rRNA-analysis datasets described in the Main text. Non-tRNA and non-rRNA reads were further aligned to *S. cerevisiae* Saccharomyces_cerevisiae.R64-1-1.ncrna.fa reference sequence (Saccharomyces_cerevisiae - Ensembl Genomes 61) and ncRNA aligned reads were processed in the same way as for rRNA. AAS score calculation and analysis was done as described previously for rRNAs.

PCA plot

Principal component analysis (PCA) was conducted on sequencing datasets from oxidized RNA samples comprising three sets of naked RNA, two sets of isolated ribosomes and three sets of in vivo oxidized RNA. The analysis was executed in R (version 4.3.2) using the prcomb() builtin function and rowVars() function, which is an integrated component of the “matrixStats” package. Statistical computations were conducted using NormGcount as previously described including only G sites without G1575 as it is naturally methylated (m^7G) and gives a high NormGcount as well as excluding G1 and G2 at the end of the RNA sequence. Visualization was done using the package ggplot2[10].

Software used in this study

Agilent MassHunter Qualitative Analysis 10.0 (Build 10.0.10305.0) was used for LC-MS/MS analysis. Excel for Microsoft 365 MSO (Version 2403 Build 16.0.17425.20176) was used for any calculations. ImageJ V5 (Version 1.52q) was used for analysis of gel images. Adobe Illustrator 2024 V5 (Version: 28.0) and BioRender.com (2024) were used for creating figures. GraphPad Prism 8.0.1 (Version 244) was used for LC-MS/MS data visualization and calculation of any statistics. Trimmomatic V0.39, Bowtie2 V2.4.2, R (version 4.3.2), using the prcomb() builtin and rawVars() function as part of the matrixStats package were used for sequencing data analysis. TapeStation Analysis Software 4.1.1 was used for total RNA electropherograms. PyMol(TM) 2.5.5 and RiboVision (Version 1.15) were used for creating figure 6.

Data availability

The sequencing data generated in this study have been deposited in the European Nucleotide Archive database under accession codes PRJEB73991 (https://www.ebi.ac.uk/ena/browser/view/PRJEB73991), PRJEB84071 (https://www.ebi.ac.uk/ena/browser/view/PRJEB84071) and PRJEB92107 (https://www.ebi.ac.uk/ena/browser/view/PRJEB92107). The remaining data generated in this study are provided in the Source Data file.

Supplementary References

1. Pospísek, M. & Valásek, L. Polysome profile analysis--yeast. *Methods in enzymology* **530,** 173–181; 10.1016/B978-0-12-420037-1.00009-9 (2013).

2. Kopietz, K. *et al.* TGT Damages its Substrate tRNAs by the Formation of Abasic Sites in the Anticodon Loop. *Journal of molecular biology* **437,** 169000; 10.1016/j.jmb.2025.169000 (2025).

3. Küpfer, P. A. & Leumann, C. J. The chemical stability of abasic RNA compared to abasic DNA. *Nucl Acids Res* **35,** 58–68; 10.1093/nar/gkl948 (2007).

4. Griffiths-Jones, S. The microRNA Registry. *Nucl Acids Res* **32,** D109-11; 10.1093/nar/gkh023 (2004).

5. Griffiths-Jones, S., Grocock, R. J., van Dongen, S., Bateman, A. & Enright, A. J. miRBase: microRNA sequences, targets and gene nomenclature. *Nucl Acids Res* **34,** D140-4; 10.1093/nar/gkj112 (2006).

6. Griffiths-Jones, S., Saini, H. K., van Dongen, S. & Enright, A. J. miRBase: tools for microRNA genomics. *Nucl Acids Res* **36,** D154-8; 10.1093/nar/gkm952 (2008).

7. Kozomara, A. & Griffiths-Jones, S. miRBase: integrating microRNA annotation and deep-sequencing data. *Nucl Acids Res* **39,** D152-7; 10.1093/nar/gkq1027 (2011).

8. Kozomara, A. & Griffiths-Jones, S. miRBase: annotating high confidence microRNAs using deep sequencing data. *Nucl Acids Res* **42,** D68-73; 10.1093/nar/gkt1181 (2014).

9. Kozomara, A., Birgaoanu, M. & Griffiths-Jones, S. miRBase: from microRNA sequences to function. *Nucl Acids Res* **47,** D155-D162; 10.1093/nar/gky1141 (2019).

10. Wickham, H. & Sievert, C. *Ggplot2. Elegant graphics for data analysis.* 2nd ed. (Springer, Dordrecht, New York, op. 2016).
